# Correcting COVID-19 vaccine misinformation in 10 countries

**DOI:** 10.1098/rsos.221097

**Published:** 2023-03-15

**Authors:** Ethan Porter, Yamil Velez, Thomas J. Wood

**Affiliations:** ^1^ School of Media and Public Affairs, George Washington University, Washington, DC 20052-0086, USA; ^2^ Department of Political Science, Columbia University, New York, NY, USA; ^3^ Department of Political Science, The Ohio State University, Columbus, OH, USA

**Keywords:** COVID-19 vaccines, misinformation, multi-country study, belief updating

## Abstract

What can be done to reduce misperceptions about COVID-19 vaccines? We present results from experiments conducted simultaneously on YouGov samples in 10 countries (*N* = 10 600), which reveal that factual corrections consistently reduce false beliefs about vaccines. With results from these 10 countries, we find that exposure to corrections increases belief accuracy by 0.16 on a 4-point scale, while exposure to misinformation decreases belief accuracy by 0.09 on the same scale. We are unable to find evidence that either misinformation or factual corrections affect intent to vaccinate or vaccine attitudes. Our findings on effect duration are less conclusive; when we recontacted participants two weeks later, we observed 39% of the initial accuracy increase, yet this result narrowly misses conventional thresholds of statistical significance (*p* = 0.06). Taken together, our results illustrate both the possibilities and limitations of factual corrections. Evidence from 10 highly diverse populations shows that exposure to factual information reduces belief in falsehoods about vaccines, but has minimal influence on subsequent behaviours and attitudes.

## 1. Introduction

Public health officials around the world have described the spread of misinformation about COVID-19 as a key factor in the continuation of the pandemic. According to a spokesperson for the World Health Organization, misinformation is ‘allowing the virus to thrive’ [1]. Such claims reflect emerging academic evidence that posits that exposure to misinformation reduces willingness to receive a vaccine [2–4]. For their part, scholars have investigated a variety of ways to counter false beliefs about COVID-19, with some signs of success [5–9]. Most efforts, however, have concentrated on one country at a time, have paid limited attention to the durability of effects, and have not reached conclusions about the relationship between reducing false beliefs and subsequent vaccine intention (see [5]). Given the global scope of the pandemic, the possibility that any effects of factual corrections may be short-lived, and the urgent need to increase vaccination, these limitations are unfortunate.

We present results from experiments conducted simultaneously on samples in 10 countries in the summer of 2021. The experiments evaluate whether factual corrections can rebut misinformation about COVID-19 vaccines and whether corrections have effects on vaccines-related attitudes and self-reported behaviours. The experiments were administered on samples in Brazil, France, Germany, India, Indonesia, Mexico, Nigeria, Peru, South Africa and the United States. For every country in our sample but Nigeria, Peru and South Africa, we were able to recontact participants to assess the durability of correction effects. The samples were collected by YouGov, which analysis shows to be a high-quality survey sample provider when compared with its peers [10,11].

With data from all 10 countries, we find that fact checks led to substantial gains in accurate beliefs, causing respondents to be less credulous toward widely circulating misinformation about COVID-19 vaccines. On a 1–4 scale, meta-analysis shows that factual corrections increased accurate beliefs by 0.16 scale points (*p* < 0.001). When we recontacted participants two weeks later, we observed 39% of the initial accuracy increase. While this suggests that the effects of fact checks are not entirely ephemeral, it is important to note that this result narrowly misses the threshold of conventional statistical significance (*p* = 0.06).

The effects of corrections were not attributable to any one country, nor only observed on a limited set of topics. Rather, we observe accuracy gains regarding a broad variety of false claims about COVID-19. Corroborating recent work [12–14], including in vaccination contexts [8,15], in no case do we detect corrections ‘backfiring’, or reducing factual accuracy. Instead, on an issue with large implications for global public health, corrections disabuse people of false beliefs.

Intriguingly, the meta-analytic effects of factual corrections are 78% larger than the effects of misinformation. While public health organizations and scholars have voiced concern about an ‘infodemic’ prolonging the pandemic [16,17], our evidence makes clear that the effects of factually accurate information on beliefs considerably—and consistently—exceed the effects of false claims. Moreover, we do not find evidence that misinformation on its own reduces intent to vaccinate or degrades vaccination attitudes. These conclusions, about the limited impact of misinformation, run contrary to prior claims about the effects of misinformation on vaccine uptake, which were based on comparatively geographically limited samples [2–4].

Corrections, however, are not a cure-all. While they improve accuracy (as shown previously in [18]), we do not observe them increasing willingness to receive a vaccine or improving attitudes toward vaccines. Instead, data collected over a wide range of countries, with distinctive economic, educational and ethnic profiles, show factual corrections increasing accurate beliefs about COVID-19 vaccines. With these data, however, we are unable to detect an effect on attitudes or behaviour. (That having been said, when considering this conclusion, it is worth keeping in mind statistical power issues, discussed below.) Our findings offer global evidence corroborating prior findings based on US-only samples [8,19] about the capacity of fact checks to improve accuracy without significantly shifting downstream outcomes.

## 2. Empirical expectations and open questions

For purposes of this study, we regard misinformation as information which has the veneer of empirical validity but runs contrary to the best evidence available at the time (a definition consistent with some of the concerns voiced by [20]). We regard factual corrections as interventions produced by journalistic and/or media organizations that challenge misinformation by presenting countervailing, verifiable evidence and arguments (similar to the definition offered in [21].

With some exceptions [18], research into the global effectiveness of factual corrections and misinformation is scant. Scanter still is global research, at the scale of this study, on the effects that factual corrections and misinformation have on COVID-19 beliefs and behaviours. Although there may be substantial differences across countries and cultures [22,23], the general weight of evidence [18,19,24–28] supports the expectation that factual corrections will reduce false beliefs about COVID-19 vaccines. For this reason, we pre-registered a hypothesis anticipating that corrections will reduce false beliefs about COVID-19 vaccines, (H1a), and a related hypothesis that factual corrections would also be effective among those sceptical of vaccines (H1b). This expectation can be contrasted with a ‘backfire effect’, where providing corrective information increases belief in the misinformation [29], a troubling phenomenon observed previously in the study of vaccines [30,31].

Yet even if corrections improve accuracy, it is possible that the effects will be short-lived. If factual corrections only affect beliefs temporarily, they would not be an especially appealing means of countering misinformation. Prior research into duration has found that, while correction effects fade [32], they do not disappear entirely [33–35]. While this evidence mostly comes from Western samples, we had no reason to believe that effects would disappear faster elsewhere. Rather, we anticipated that correction effects would fade but remain detectable about two weeks after initial exposure (H2).

From both public health and scholarly perspectives, the effects of fact checks and misinformation on vaccine attitudes and behaviours among the non-vaccinated are especially important. As it may be the case that exposure to either factual corrections or misinformation can affect willingness to take a vaccine, we pre-registered two research questions concerning effects on vaccine intent among the non-vaccinated (RQ1a-RQ1b). On the one hand, prior evidence indicates that the effects of factual corrections are mostly limited to factual belief accuracy [8,19]. On the other hand, this evidence comes from single-country samples. So far as we know, little global evidence exists on the relationship between factual corrections and subsequent attitudes and behaviours (as distinct from belief accuracy). Unlike evidence on the relationship between factual corrections and belief accuracy, for which the conclusions are clear, evidence on the relationship between corrections and subsequent behaviour is more uncertain. Our uncertainty led us to investigate this matter as research questions.

We leveraged our international scope to evaluate how certain pre-treatment characteristics affect responses to misinformation and factual corrections. Although scholars have repeatedly identified responses to the Cognitive Reflection Test (CRT) as shaping the effects of misinformation [36], prior global research on factual corrections and misinformation has not examined whether this pattern holds globally. Given the absence of cross-national evidence on this question, we pre-registered a research question to evaluate it (RQ2). Similarly, we pre-registered research questions concerning the relationship between treatment effects and pre-treatment measures of ideology (RQ3) and conspiracism (RQ4), which prior research indicates may shape beliefs about COVID-19 [37,38]. (We also pre-registered a research question (RQ5) concerning the self-reported amount of money non-vaccinated individuals would require to receive a vaccine; that result is beyond the substantive scope of the present paper and will be addressed at a later date). Finally, because we tested two global false claim-correction pairs, common across all countries included, and country-specific pairs, we pre-registered a research question (RQ6) about differences in effects, if any, between global and country-specific pairs.

## 3. Design

The experiments were conducted simultaneously across 10 countries from June to July 2021. We prioritized locations that scored high on vaccine scepticism prior to the roll-out of the vaccine [39], while also balancing the need to represent different global regions (e.g. the Global South). Moreover, as fact-checking is increasingly a global phenomenon, we sought to collect data in contexts where fact-checking organizations were present, so we could present participants with credible experimental stimuli. Participants in each country began by answering key demographic questions, providing answers to the Cognitive Reflection Test (CRT), reporting their ideology, and responding to questions about their vaccination status along with general attitudes toward vaccines and conspiracies, leveraging previously used batteries. The complete text of all items can be found in the electronic supplementary material, appendix.

Participants were then enrolled in three within-subjects trials. In each trial, participants could be assigned to a control condition, in which they answered only outcome questions; a misinformation condition, in which they were exposed to misinformation before answering outcome questions; or a misinformation and factual correction condition, in which they were exposed to misinformation and a factual correction before answering outcome questions. After all three trials, participants were extensively debriefed about any misinformation they encountered during the study.

In each country, two of the three trials concerned identical global false claims, while the remaining trial concerned a country-specific false claim. Our global false claims, studied in all 10 countries, involved claims that the Gates Foundation profits from the distribution of vaccines and that mRNA vaccines modify DNA. Our country-specific items touched on a wide variety of false claims, from one in South Africa alleging that mRNA vaccines altered genetic material to one in Peru that vaccines contained new variants of the disease. (Summaries of all items can be glimpsed in figure 2 and electronic supplementary material, table S17; the appendix contains the complete text.) The order of items in each country was randomized; respondents could have seen the country-specific and global items in any order.

To maximize realism, we relied on factual corrections produced by fact-checking organizations that were signatories to the standards of the International Fact-Checking Network (IFCN). While the global corrections were translated as necessary, the fact checks were otherwise not modified in any way, and are similar to fact checks tested in other research [32]. Whenever possible, we presented the misinformation in its original format (e.g. by presenting viral videos). For the two global items, we relied on factual corrections produced by PolitiFact, translating as necessary. This approach maximized ‘mundane realism’, as it ensured that our stimuli would resemble stimuli subjects could encounter in the real world [40]. However, as subjects were also compelled to be exposed to stimuli (e.g. participants assigned to video stimuli could not immediately move forward), our study lacked what [40] terms ‘psychological realism’, insofar as it did not approximate the conditions in which people encounter fact checks. The selection of both global and country-specific corrections was made after using Facebook CrowdTangle data to identify popular misinformation about COVID-19 vaccines in each country and then matching that misinformation to factual corrections produced by an IFCN signatory organization. While we are confident that the tested misinformation topics were popular at the time, it is important to note that our stimuli selection was necessarily conditional on the existence of accompanying factual corrections.

**Figure 1.**
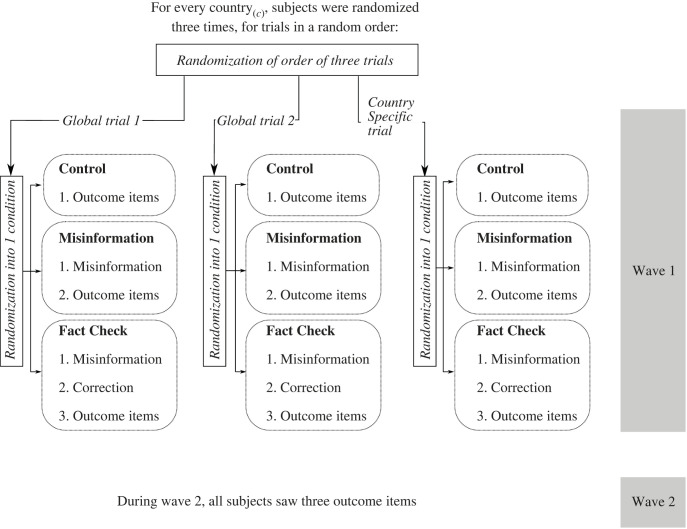
Diagrammatic summary of experimental design. All countries participated in wave 1. All countries *except* Nigeria, Peru and South Africa, also participated in wave 2.

Figure 1 provides a diagrammatic summary of this design as it was carried out across countries. For purposes of illustration, consider how a participant in France could have experienced the three trials. After answering pre-treatment questions, she would have been enrolled in her first trial. She could first have been assigned to a trial focused on a global item, concerning the Gates Foundation. In that trial, she could have been assigned to control and asked to respond to a question about whether the Foundation was profiting from the vaccine. After this first trial—and only after this first trial—she would then answer questions about vaccine uptake and attitudes. For her second trial, she could have been assigned to France's country-specific item, which involved a claim that vaccinated people were more contagious than non-vaccinated people. If she were assigned to a misinformation condition, she would have seen a video advancing that claim, and then asked outcome questions. Finally, for her third trial, she would have been assigned to the remaining global item, about mRNA vaccines. If she had been assigned to the misinformation and factual correction condition, she would have seen misinformation, then a factual correction, and finally answered outcome questions. At the end of the three trials, she would receive a fact check addressing the France-specific misinformation, since she was in the misinformation condition during her second trial.

To assess effects on factual beliefs, at the end of each trial, participants assessed the accuracy of the corresponding false claim on a 1–4 accuracy scale ranging from ‘not at all accurate’ to ‘very accurate’. For example, for the country-specific item in France, respondents were asked how accurate the following statement was: ‘According to scientists, vaccinated people are more contagious than those who have not received a Covid vaccine’. This approach mirrors prior outcome measurement in this literature [12]. To measure effects on intent to vaccinate and attitudes toward vaccines, we relied on batteries used previously and asked participants after the first trial only how likely they would be to take a vaccine once they were eligible to receive it (on a 1–7 scale), and to agree-disagree with statements about their attitudes toward vaccines (each on a 1–5 scale). The complete text of the survey can be found in the electronic supplementary material, appendix.

In the countries for which we were able to recontact participants, we began a second wave of data collection roughly two weeks after initial contact. In this second wave, participants answered the identical set of outcome questions they had answered in the prior wave. We did not remind participants of their earlier treatment assignments in this follow-up wave.

## 4. Results

**Figure 2.**
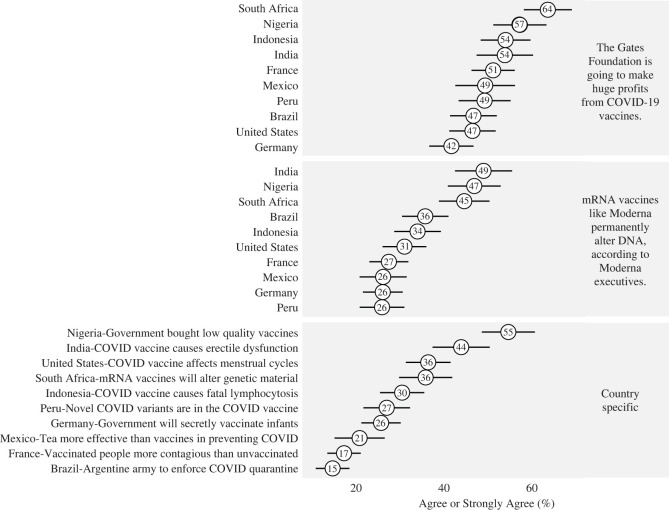
Belief in false claims among control participants, with 95% confidence intervals.

Descriptive and balance statistics, including information on treatment assignment in each country, can be found in electronic supplementary material, table S1. Across all 10 countries (*N* = 10 600), we were well balanced across conditions. In addition, even as some evidence suggests that false beliefs about COVID-19 are not especially widespread around the world [41,42], our tested misinformation items proved largely salient in the studied populations. In figure 2, we visualize level of agreement with each false claim among participants who were exposed to neither the misinformation nor fact-checking stimuli (that is, among participants in the control group for that given item). The figure shows country-by-country belief, for both country-specific and global items. On average, 51% of control participants agreed that Gates Foundation was set to make enormous profits from the COVID-19 vaccines; 36% agreed that mRNA vaccines permanently alter DNA. On average, across items, 31% of control participants believed their country-specific claim.

**Figure 3.**
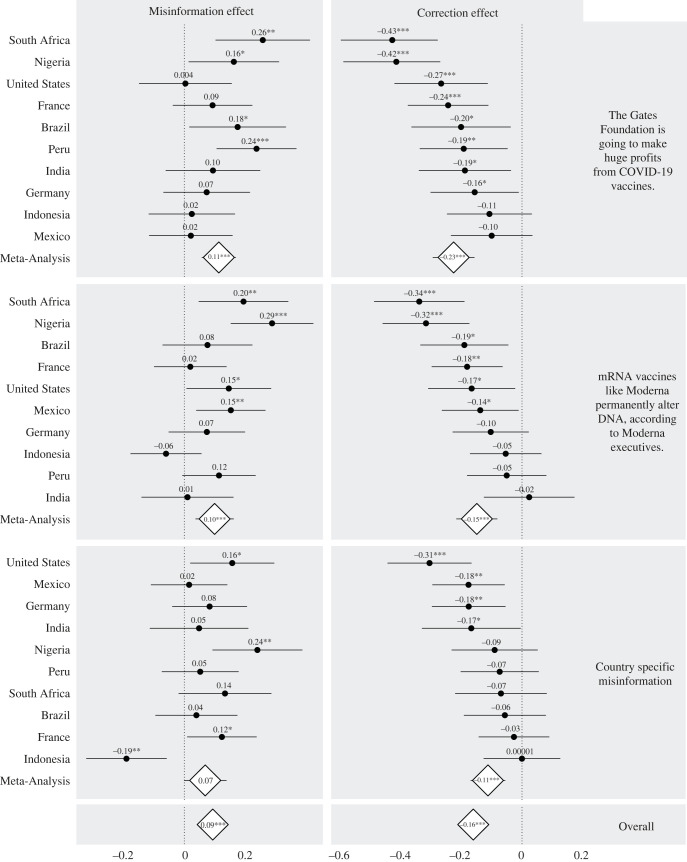
Misinformation and correction effects, by corrected issue, at wave 1. Accuracy is measured on a 4-point scale, with higher values indicating greater accuracy. Estimates inside diamonds report meta-analytic summaries. Corresponding regression results can be found in electronic supplementary material, tables S2–S5. The difference between the Gates Foundation and country-specific fact-check meta-analytical estimate is the only statistically significant difference we can detect (*p* = 0.01) between global and country-specific items. In all other cases, there is no statistically distinguishable difference between the global and country-specific fact checks or misinformation meta-analytical estimates.

Targeting these false claims, factual corrections improved belief accuracy. In figure 3, we depict misinformation and correction effects in each country and offer meta-analytic estimates of the effects of both corrections and misinformation. While misinformation degraded factually accurate beliefs by 0.09 on a 4-point scale (*p* < 0.001), factual corrections improved accuracy by 0.16 on the same scale (*p* < 0.001). All tests are two-sided. These effects are robust to the Bonferroni correction procedure for multiple comparisons, as well as other conventional correction procedures. (electronic supplementary material, table S5 contains the results of the meta-analysis, while more information on multiple comparisons can be found in appendix section 9). Across a wide array of countries and substantive topics, the effects of misinformation on factual accuracy proved smaller than the effects of factual corrections. The effects of corrections and misinformation by country, on country-specific and global items, can be found in electronic supplementary material, tables S2–S4.^1^ Electronic supplementary material, figure S23 displays effects by control-group standard deviations. As an additional robustness check, we compare our ordinary least squares (OLS) regression coefficients to ordinal probit coefficients. They are correlated at more than 0.99; electronic supplementary material, figure S8 displays results.

Yet even as corrections improved accuracy, we do not find evidence that they increased intent to vaccinate (*p* = 0.43; electronic supplementary material, table S6). This finding extends prior conclusions about the capacity of fact checks to improve accuracy without affecting related attitudes [8,19,35] to the global stage. However, what is observed in a smaller set of countries does not always extend to a larger part of the world. Unlike [2], which only examines two countries, as well as other work [3,4], we find no evidence across 10 countries that misinformation reduces intent to vaccinate (*p* = 0.72; electronic supplementary material, table S6). Nor do we find evidence that either fact checks or misinformation change broader attitudes toward vaccines in either direction (*p* = 0.98, *p* = 0.74, respectively; electronic supplementary material, table S7). While we fail to reject the null hypothesis, we do not have sufficient power to rule out conventionally small effects in either direction. Our design had 80% power to detect effects up to 0.055 standard deviation units for the vaccine attitudes scale and 0.27 standard deviation units for the vaccine intention scale.

**Figure 4.**
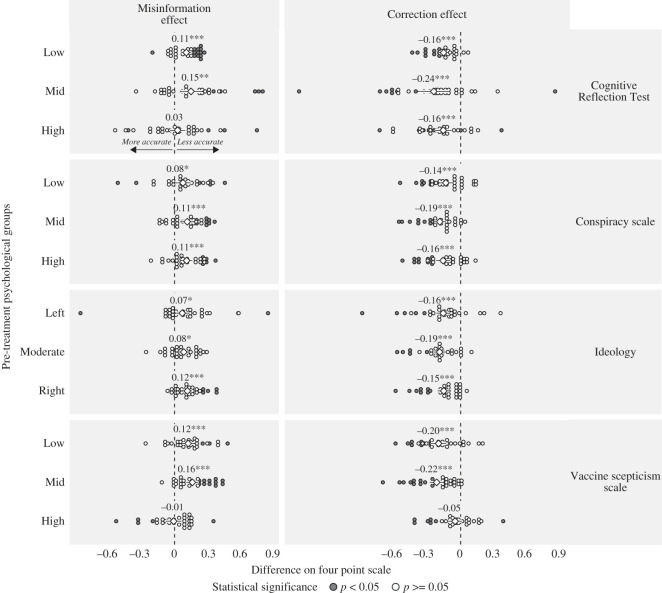
Misinformation and correction effects, by pre-treatment performance on the Cognitive Reflection Test, conspiracism and vaccine scepticism, with 95% confidence intervals.

Pre-treatment levels of vaccine scepticism shaped the effects of fact checks and misinformation on belief accuracy. In figure 4, we display treatment effects on belief accuracy, for both misinformation and fact checks, grouping participants by pre-treatment ideology, vaccine scepticism, responses to the Cognitive Reflection Test and penchant for conspiracism. As the second row of figure 4 shows, participants who were in the top tercile of vaccine scepticism were least affected by corrections in the direction of greater accuracy. At the same time, however, the most sceptical subjects were not detectably affected by misinformation. As figure 4 also indicates, we observe less evidence of effect heterogeneity by the remaining three pre-treatment measures than we do by vaccine scepticism. All levels of ideology are affected by corrections and misinformation in the same directions. The same is largely true for pre-treatment conspiracism and responses to CRT (although we do not find evidence that low levels of CRT are associated with susceptibility to misinformation, as anticipated by [36]). We tested other potential sources of heterogeneity as well, including those related to COVID-19 at the time of the study (e.g. vaccination and infection rates by country), and political and economic factors, such as economic inequality, trust in government and governing ideology. The results of these exploratory analyses, which were not pre-registered, can be found in the electronic supplementary materials, as figures S4 and S5.

**Figure 5.**
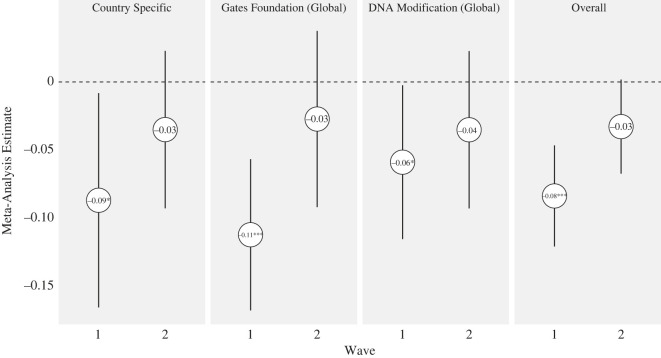
Estimates of difference between fact-check condition and pure control in Wave 1 and Wave 2, with 95% confidence intervals. Estimates are based on the seven countries for which we have Wave 2 data (i.e. US, France, Germany, Brazil, Mexico, Indonesia and India).

We now assess the durability of fact checks relative to pure control. Since those exposed to any misinformation received a fact check at the end of Wave 1, we can only assess durability relative to respondents in the pure control condition. This probably reduces our effect size, since we cannot adjust for the priming or learning effect associated with reading about misinformation in the context of a fact check. Figure 5 displays results. Aggregating across all available fact checks, while we see that the meta-analytical fact-check effect in Wave 1 is 0.08 scale points (*p* < 0.001) on a 1–4 point scale in Wave 1, the effect is reduced to 0.03 points in Wave 2, very narrowly missing the threshold for statistical significance (*p* = 0.06, two-tailed). (Consult electronic supplementary material, table S14 for complete durability results.)

Finally, given the wealth of covariates and items tested, and the global scope of this project, we conducted an exploratory variation of a specification curve analysis. Specification curves evaluate the possible universe of reasonable models, estimating a separate model for every combination of possible analytic choices, to determine if estimated effects are responsive to these choices [43]. Our specification curve analysis varied four classes of modelling choices: the specific controls used in a model, which misinformation treatment was corrected, which countries were included in our analysis, and finally, using subsets of respondents as defined by their pre-treatment covariates. To make effects comparable across countries, we only focused on the global items. This analysis makes clear that factual backfire is exceedingly rare: of 1920 models estimated, in only 24 do we observe significant factual belief degradation. The specification curve can be found in electronic supplementary material, figure S1. When we replicate this exercise for misinformation effects, we find that the meta-analytic estimate of misinformation effects, across all possible models, is less than half the size of the comparable estimate for correction effects (electronic supplementary material, figure S2).

## 5. Discussion

With data from a wide array of countries and continents, we find that correcting misinformation about COVID-19 improves factual accuracy. This evidence both substantially expands the scope of available cross-national evidence on the effectiveness of factual corrections and demonstrates that these effects are robust to the COVID-19 pandemic. Furthermore, unlike prior work on the global effects of factual corrections [18], we assess whether corrections also affect downstream attitudes and behaviours. On the critically important question of vaccine uptake, we find no evidence that corrections of misinformation increase willingness to receive a vaccine (corroborating [44], but deviating from [45]). Echoing voluminous research on inconsistencies between beliefs and behaviours (e.g. [46]), our findings extend prior conclusions about fact checks that had been based on single-country samples [8,19]: corrections improve accuracy, but we are unable to detect an effect on attitudes or behaviour.

Our results may be read to suggest that misinformation's effects are more circumscribed than sometimes feared. After all, the effects of misinformation on factual beliefs are smaller than the effects of fact checks. In absolute terms, our meta-analysis shows that the effects of factual corrections on belief accuracy are 78% larger than the effects of misinformation. In addition, misinformation widely circulating around the world has no apparent influence on willingness to receive a vaccine (though again, we urge readers to keep statistical power in mind when interpreting this result). This finding should temper strong claims about the extent to which misinformation is responsible for vaccine scepticism. Misinformation is probably one of many reasons that people are vaccine-sceptical. None of this is to say that misinformation on its own does not cause harms. On the contrary, as we demonstrate, around the world, misinformation degrades factually accurate knowledge about COVID-19. It is possible that repeated exposure to misinformation, delivered over a longer time period, would have effects on vaccine uptake larger than those we observe here.

With that said, why do our results deviate from prior researchers who have observed misinformation degrading vaccination beliefs and behaviours [2–4]? Several potential explanations come to mind. First, the tested misinformation may have been too far afield from those factors that truly affect vaccination-related attitudes. For example, participants may have responded to corrections about COVID-19 vaccines and fertility by becoming more accurate—but misinformation about infertility may simply not have played a role in participants' vaccination-related behaviours and attitudes. Second, pre-treatment effects [47], related to participants having lived through a pandemic for more than a year at the time we fielded the study, may have blunted the impact of tested misinformation. Had we fielded the study immediately after the emergence of a COVID-19 as a global pandemic, perhaps we would have observed larger misinformation effects. In the time elapsed since the pandemic's start, attitudes toward vaccination may already have been affected by misinformation to the point where exposure to one additional false item was unlikely to make a difference. This explanation suggests a pronounced role for attitude strength (see [48] for a review), and underscores that more attention should be paid to understanding the dose–response relationship that misinformation has with related attitudes and behaviours.

Although the effects of misinformation can be exaggerated, the effects of factual corrections are also limited. Perhaps most importantly, we are unable to find that they have any impact on willingness to receive a vaccine or attitudes toward vaccines. As with misinformation, this particular limitation may be related to dosage; larger quantities of factual corrections may increase vaccine intent. Nor are the effects of corrections strongly durable. Again, dosage may be a culprit. Speculatively, we surmise that the effects of factual corrections are at least as durable as accuracy nudges, another established tool that helps combat misinformation [49]. Factual corrections are more demanding of participants' time, and that additional time may leave a longer-lasting impression. Finally, factual corrections are not uniformly effective around the globe.

The broader theoretical implications of these findings deserve further investigation. At first blush, they would appear to conflict with a motivated-reasoning account, wherein directional goals outpace accuracy goals [50]. That corrections improve accuracy generally across our 10 countries, and that the effects of corrections on accuracy exceed the effects of misinformation on the same outcome, would suggest that accuracy goals are more appealing than sometimes assumed. However, the inability of corrections to change related attitudes and behaviours suggests that accuracy goals may matter most when accuracy itself is the outcome at stake; directional goals may be more powerful otherwise. Indeed, the accuracy increases we observe may reflect the weakness of accuracy goals in general, with respondents regarding factual accuracy as too unimportant an outcome to marshal the effort required to counter-argue the facts (as argued in [26]). Ultimately, our findings may be reconcilable with a standard Bayesian updating account, as [51] shows can be done with motivated reasoning. For now, the minimal ability of this paper to respond to these theoretical concerns stands out as one limitation.

The country-by-country variation is worthy of additional investigation. Some of the variations across the country-specific items may be attributable to differences in fact-checking quality across countries; differences in the content of misinformation across countries; differences in outcome measurement; and differences in the correctability of popular misinformation topics. Recall that the country-specific fact checks were produced by in-country fact-checking organizations, while the global fact checks were all originally produced by one organization, and evaluated using the same outcome questions. Country-by-country differences for the country-specific correction and misinformation effects may be associated with the substantive relationship between the tested stimuli and the outcome measures.

Intriguingly, in electronic supplementary material, figure S3, we present evidence showing that, by country, country-specific correction effects are not meaningfully predicted by correction effects targeting global misinformation items. This suggests that idiosyncrasies of individual country-specific fact checks are not solely responsible for differences in correction effects across countries. More research is needed on the role that latent features of the texts of fact checks, underlying misinformation, and outcome measurement may play in the variation of their effects.

Several additional limitations of this study suggest avenues for future research. First, while we relied on real-world stimuli, thereby heightening realism [40], we are sceptical that people in the outside world read fact checks in as detailed a manner as people enrolled in surveys, limiting the ecological validity of these findings. Second, our inability to test larger doses of fact checks and misinformation may be arresting our capacity to identify the effects of both on vaccine-related outcomes. Third, our cross-country approach leaves us reliant on country-by-country variations in misinformation environment and correction quality. We look forward to future research that carefully inspects the potential sources of heterogeneity, including but not limited to those described in electronic supplementary material, figures S4 and S5. Finally, we look forward to future work that resolves the duration question. While readers may regard our duration findings as weak but inconclusive evidence in favour of duration, meta-analyses will probably be able to shed more definitive light on the subject.

Scholars, policymakers and social media companies investigating ways of curbing COVID-19 misinformation should study whether increasing dosage of fact checks affects durability and vaccine hesitancy; the potential of more ecologically valid designs; and the extent to which quality of fact checks and characteristics of misinformation intersect to shape correction effects. For now, however, our evidence makes clear that false beliefs about COVID-19 vaccines can be rebutted around the world through the use of factual corrections, but neither misinformation nor corrections appear to play a large role in willingness to receive a vaccine.

## Data Availability

The code and data can be accessed here: https://dataverse.harvard.edu/dataset.xhtml?persistentId=doi%3A10.7910%2FDVN%2FOFPUAD. The data are provided in electronic supplementary material [52].

## Footnotes

1 In eight of the 10 countries, meta-analysis of the three trials shows that corrections had a significant effect on belief accuracy. The exceptions are India and Indonesia. The results of this exercise can be glimpsed in the electronic supplementary material, figure S21. This analysis was not pre-registered.
